# Decreased Serum 25-(OH)-D Level Associated With Muscle Enzyme and Myositis Specific Autoantibodies in Patients With Idiopathic Inflammatory Myopathy

**DOI:** 10.3389/fimmu.2021.642070

**Published:** 2021-04-15

**Authors:** Zhen Yu, Hao Cheng, Yuying Liang, Tingting Ding, Chenglan Yan, Chong Gao, Hongyan Wen

**Affiliations:** ^1^ Department of Rheumatology, Shanxi Medical University Second Affiliated Hospital, Taiyuan, China; ^2^ Department of Pathology, Brigham and Women’s Hospital, Harvard Medical School, Boston, MA, United States

**Keywords:** idiopathic inflammatory myopathy, 25-hydroxy-vitamin D, muscle enzyme, myositis specific autoantibodies, lymphocyte subsets

## Abstract

**Objectives:**

To determine whether there is serum vitamin D deficiency and the low levels of serum vitamin D are correlated with serological and immunological indexes in patients with idiopathic inflammatory myopathy (IIM).

**Methods:**

A total of 63 newly diagnosed patients with IIM, and 55 age- and sex- matched healthy controls were enrolled. Serum levels of 25-(OH)-D were measured by enzyme-linked immunosorbent assay. The correlations of 25-(OH)-D levels with disease indicators and T cell subsets were analyzed.

**Result:**

The levels of serum 25-(OH)-D in IIM were significantly lower than those in healthy controls (9.36 ± 5.56 vs 26.56 ± 5.37 ng/ml, p<0.001). The levels of serum liver enzyme ALT and AST and muscle enzyme CK, CKMB, LDH and HBDH were elevated as deficiency of vitamin D. In addition, the serum 25-(OH)-D levels were negatively correlated to ALT (r = -0.408, p = 0.001) and AST (r = -0.338, p = 0.007). The 25-(OH)-D levels in IIM patients in presence of anti-Jo-1 were significantly lower than those in patients without anti-Jo-1 (5.24 ± 3.17 vs 9.32 ± 5.60 ng/ml; p = 0.037). Similar results were found in patients with or without anti-Mi-2 antibody. The serum 25-(OH)-D levels were positively associated with total T (r = 0.203, p = 0.012) and Treg cells (r = 0.331, p = 0.013). The patients with deficient levels of vitamin D were more likely to have heliotrope, gastrointestinal and liver involvement.

**Conclusions:**

Vitamin D deficiency existed in IIM patients, which was significantly correlated with muscle enzyme, presence of anti-Jo-1 and anti-Mi-2 antibody, and the absolute numbers of total T and Treg cells in IIM. It is suggested that vitamin D may play an important role in the immunological pathogenesis of IIM.

## Introduction

Idiopathic inflammatory myopathy (IIM) covers a complex group of systemic autoimmune diseases which has proximal muscle weakness, fatigue, and elevated serum muscle enzymes, together with myofiber degeneration or fibrosis and mononuclear cell infiltration represent. There are four main clinical subtypes: polymyositis (PM), dermatomyositis (DM), immune-mediated necrotizing myopathy (IMNM) and sporadic inclusion body myositis (sIBM) (1).

Vitamin D is a class of fat-soluble vitamins that belong to the steroid group. It is generally believed that vitamin D plays an important role in calcium and phosphorus metabolism and in the regulation of bone formation and resorption. 1,25-dihydroxycholecalciferol [1,25(OH)_2_D_3_], which was the active form of vitamin D, could influence the proliferation and differentiation of many immune cells and regulate the expression of cytokines and autoantibodies to play the role of immune regulation (2). Recent studies have related vitamin D deficiency with several autoimmune diseases, including rheumatoid arthritis (RA) (3), multiple sclerosis (MS) (4), and systemic lupus erythematosus (SLE) (5). It is reported that 1,25(OH)_2_D_3_ plays a crucial role in immune regulation effect in these autoimmune diseases. Payam Azali et al. (6) found vitamin D deficiency is common in IIM patients, but the associations between vitamin D deficiency and clinical parameters in IIM remain is unclear.

In this study, we investigated the levels of 1,25(OH)_2_D_3_, the clinical indicators and laboratory examinations from 63 IIM patients and analyzed the correlations of 1,25(OH)_2_D_3_ with clinical and laboratory indexes in patients with IIM.

## Materials and Methods

### Patients and Enrolment Criteria

This was a cross-sectional retrospective observational case-control study. A total of 63 newly diagnosed and untreated IIM patients from rheumatologic clinic of the Shanxi Medical University Second Affiliated Hospital were enrolled from January 2016 to June 2018. Seven patients (11%) were classified as PM, 49 (78%) as DM and 7 (11%) as connective tissue myositis (CTM). The CTM subgroup included patients meeting the criteria for myositis and another autoimmune disease, including 2 with rheumatoid arthritis, 2 with systemic sclerosis and 3 with primary Sjögren’s syndrome. Diagnosis of IIM was made according to the classification criteria of Bohan and Peter (7, 8), with the exception of non-inflammatory myopathies such as muscular dystrophy and metabolic myopathy. Patients receiving or who had ever received vitamin D, corticosteroids, disease-modifying anti-rheumatic drugs (DMARDs), or tumor necrosis factor antagonists were excluded. The 55 age- and sex-matched healthy volunteers without history of rheumatic immune system disease or family history were received physical examination in the Physical Examination Center of our hospital for the same period (**Table 1**). Serum samples were taken, the clinical and laboratory indexes and antibodies were detected using freshly blood samples at the same time. Our study was approved by the Ethics Committee of the Shanxi Medical University Second Affiliated Hospital and all candidates have signed informed consent.

**Table 1:** Demographic and clinical indicator data of IIM patients and healthy controls (HCs).

Characteristics	HCs	IIM patients	p Value
Age (years)	52.38 ± 13.01	53.73 ± 11.58	0.552
Female	82% (45/55)	83% (52/63)	0.919
Male	18% (10/55)	17% (11/63)	0.919
Height (m)	1.59 ± 0.08	1.60 ± 0.07	0.576
Weight (kg)	55.29 ± 12.53	62.09 ± 10.47	**0.002**
BMI (kg/m^2^)	21.56 ± 3.81	24.15 ± 3.87	**0.001**
Disease duration (years)	–	1.40 (0.32, 5.51)	–
ESR	7.90 ± 4.85	50.05 ± 33.32	**<0.001**
CRP	3.04 ± 1.80	36.10 ± 66.45	**<0.001**
WBC (*10^9^/l)	6.14 ± 1.61	7.51 ± 3.62	**0.011**
Hb (g/l)	136.44 ± 17.73	120.95 ± 17.84	**<0.001**
PLT (*10^9^/l)	248.31 ± 60.95	241.32 ± 103.50	0.662
LY (*10^9^/l)	2.13 ± 0.74	1.32 ± 0.72	**<0.001**
Anti-Jo-1	–	6% (4/66)	–
Anti-Mi-2	–	5% (3/66)	–

Results are given as mean ± SD, M (P_25_, P_75_) or percentage (count/total). BMI, Body Mass Index; ESR, Erythrocyte sedimentation rate; CRP, C-reactive protein; WBC, White blood cell; Hb, Hemoglobin; PLT, Platelet; LY, lymphocyte; Anti-Jo-1, anti-histidyl-tRNA synthetase. Bold values mean statistical significance.

### Study Design

Demographic data like gender, age, and disease duration (time elapsed since symptom onset) were included. The serum levels of 25-hydroxy-vitamin D [25-(OH)-D] were measured by enzyme-linked immunosorbent assay (ELISA). Patients were categorized into four groups by the serum 25-(OH)-D levels: extremely deficient (25-(OH)-D: <10 ng/ml), deficient (25-(OH)-D: 10.1-20 ng/ml), insufficient (25-(OH)-D: 20.1-30 ng/ml), sufficient (25-(OH)-D: > 30 ng/ml) (9).

Erythrocyte sedimentation rate (ESR) was analyzed by the Westergren method. C-reactive protein (CRP), complement C3 (C3), complement C4 (C4), blood urea nitrogen (BUN), creatinine (Cr), alanine aminotransferase (ALT), aspartate aminotransferase (AST), creatine kinase (CK), creatine Kinase Isoenzyme-MB (CKMB), lactate dehydrogenase (LDH), hydroxybutyrate dehydrogenase (HBDH) were detected with automatic biochemical analyzer. The myositis specific autoantibodies (Anti-Jo-1, Anti-SRP and Anti-Mi-2), myositis associated autoantibodies (Anti-Ro52) and other autoantibodies (ANA, Anti-SSA, Anti-SSB and Anti-ENA) were detected by Western blotting.

Immunophenotypes of lymphocyte subsets were determined by a FACSCalibur flow cytometer (BD Biosciences, Franklin Lakes, NJ, USA). All immunofluorescence antibodies were purchased from BD Biosciences. Peripheral blood samples (5 ml) were collected from each patient in EDTA anticoagulant tubes according to the manufacturer’s recommendations (BD Biosciences, Franklin Lakes, NJ, USA). Peripheral blood mononuclear cells (PBMCs) were separated by Ficoll-hypaque density gradient centrifugation. Within 30 minutes, 20 μl fluorescent antibody were added to an 80 μl blood sample and incubated at room temperature in the dark for 15 minutes. Then, 2 ml freshly prepared fixation/permeabilization solution was added, mixed, and incubated at 4 °C for 10 minutes, followed by washing with PBS and analyzing by flow cytometry and MultiSET software (BD Biosciences). The absolute numbers of the cell subsets were determined: total T cell [CD3+CD19-; normal range (NR): 955 ~ 2860/μl], total B (CD3-CD19+; NR: 90 ~ 560/μl), natural killer (NK) (CD3-/CD16+CD56+; NR: 150 ~ 1100/μl), CD4+ T (Th) (CD3+CD4+; NR: 378 ~ 1245/μl), CD8+ T (OCD3+CD8+; NR: 128 ~ 993/μl), T helper 1 (Th1) (IFN-γ; NR: 5.52 ~ 182/μl), T helper 2 (Th2) (IL-4; NR: 4.04 ~ 21/μl), T helper 17 (Th17) (IL-17; NR: 3.07 ~ 14/μl), and regulatory T (Treg) cells (CD4+CD25+Foxp3; NR: 17.7 ~ 54.2/μl). The ratios of Th1 versus Th2 cells (Th1/Th2) and Th17 versus Treg cells (Th17/Tregs) were calculated (NR: 0.73 ~ 18.50, 0.09 ~ 0.47).

Organ involvement was defined as the presence of other diseases at the time of the diagnosis of IIM. Lung involvement including dyspnea on exertion, interstitial lung disease, dysphonia. Cardiac involvement including arrhythmia, heart failure. Gastrointestinal involvement including dysphagia and regurgitation. Liver involvement including liver enzymes increased. Kidney involvement including increased creatinine, proteinuria, microscopic hematuria and renal failure.

### Statistical Analysis

Dichotomous variables were expressed as percentages and absolute frequencies, and continuous features were expressed as mean ± standard deviation (SD) or median four quantile method [*M* (*P_25_*, *P_75_*)]. Categorical variables were compared using χ^2^ Test. For continuous features, two samples were compared with independent samples *t*-test, and the Kruskal-Wallis test was used for comparison between several groups. The Spearman rank correlation coefficient was performed to examine associations and estimate the contributing factors affecting serum 25-(OH)-D levels. *P* value of less than 0.05 was considered statistically significant. Statistical analyses were performed by SPSS version 23.0 (IBM Corp, Armonk, NY, USA) and GraphPad Prism version 8.01.

## Results

**Figure 1:**
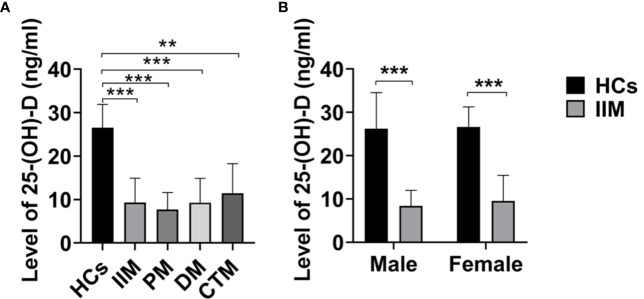
The levels of 25-(OH)-D (ng/ml) in IIM patients and HCs. **(A)** The levels of 25-(OH)-D in IIM and its subgroups were significantly lower than those of HCs. **(B)** The levels of 25-(OH)-D in IIM were significantly lower than those of HCs in male and female. HCs, Healthy controls; IIM, idiopathic inflammatory myopathy; PM, polymyositis; DM, dermatomyositis; CTM, connective tissue myositis. **p < 0.01, ***p < 0.001 by independent samples t-test.

The mean serum 25-(OH)-D levels of IIM patients were significantly lower than those of HCs (9.13 ± 5.29 vs 22.86 ± 5.38; p<0.001; **Figure 1A**) and those in male or female patients was also significantly lower than those in healthy males or females, respectively (**Figure 1B**). Furthermore, the serum vitamin D levels in all IIM subgroups were significantly lower than those of HCs (**Figure 1A**). The patients were categorized into four groups based on the serum 25-(OH)-D levels (**Table 2**). Of 63 IIM individuals and all IIM subgroups, the overwhelming majority were extremely deficient in 25-(OH)-D levels <10 ng/ml, while almost all HCs had vitamin D with 25-(OH)-D levels over 20 ng/ml.

**Table 2:** Number (%) of IIM patients and controls with extremely deficient, deficient, insufficient and Sufficient serum levels of 25-(OH)-D.

	Extremely deficient (< 10 ng/ml)	Deficient (10.1-20 ng/ml)	Insufficient (20.1-30 ng/ml)	Sufficient (>30 ng/ml)	Total
Controls	1 (1.8%)	8 (14.6%)	22 (40%)	24 (43.6%)	55 (100%)
IIM	42 (66.7%)	17 (27.0%)	4 (6.3%)	0 (0%)	63 (100%)
PM	5 (71.4%)	2 (28.6%)	0 (0%)	0 (0%)	7 (11.1%)
DM	32 (65.3%)	14 (28.6%)	3 (6.1%)	0 (0%)	49 (77.8%)
CTM	5 (71.4%)	1 (14.3%)	1 (14.3%)	0 (0%)	7 (11.1%)

**Figure 2:**
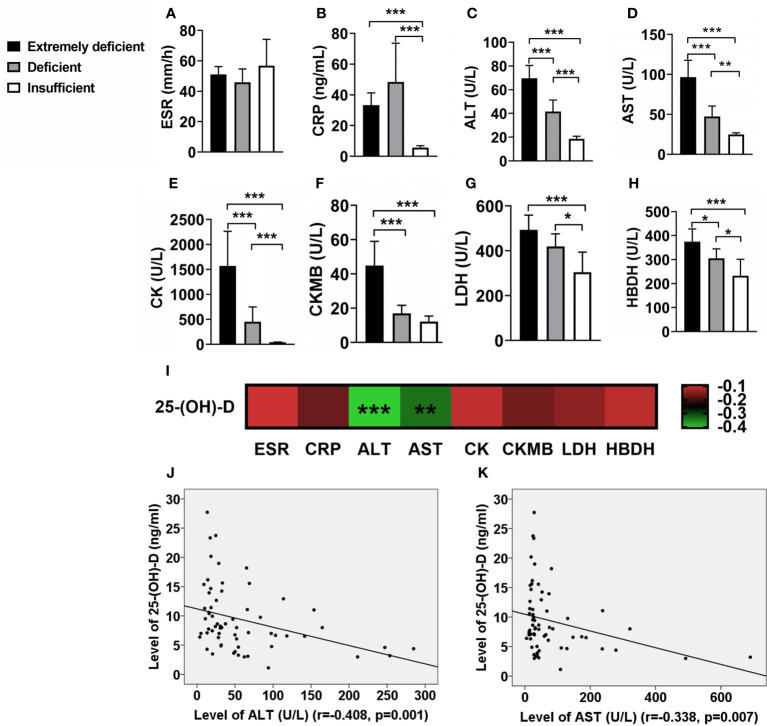
The associations of vitamin D levels with inflammatory biomarkers in IIM patients. **(A)** ESR has no significant correlation with vitamin D. **(B)** The level of CRP in patients with deficient vitamin D were significantly higher than those of patients with insufficient vitamin D. **(C–H)** As the degree of vitamin D deficiency increased, the levels of serum muscle enzymes elevated. **(I)** The associations of 25-(OH)-D levels with inflammatory biomarkers in IIM patients. **(J, K)** The serum 25-(OH)-D levels were negative correlated with ALT and AST. *p < 0.05, **p < 0.01, ***p < 0.001.

In IIM patients with different levels of vitamin D, the ESR had not significantly difference (**Figure 2A**), but the CRP in IIM with deficient vitamin D was significantly higher than those in IIM with insufficient vitamin D (**Figure 2B**). Interesting, the levels of serum liver enzyme ALT and AST and muscle enzyme CK, CKMB, LDH and HBDH were elevated as deficiency of vitamin D became severer (**Figures 2C–H**). In addition, the serum 25-(OH)-D levels were negative correlated with ALT (r = -0.408, p = 0.001; **Figures 2I, J**) and AST (r = -0.338, p = 0.007; **Figures 2I, K**).

**Figure 3:**
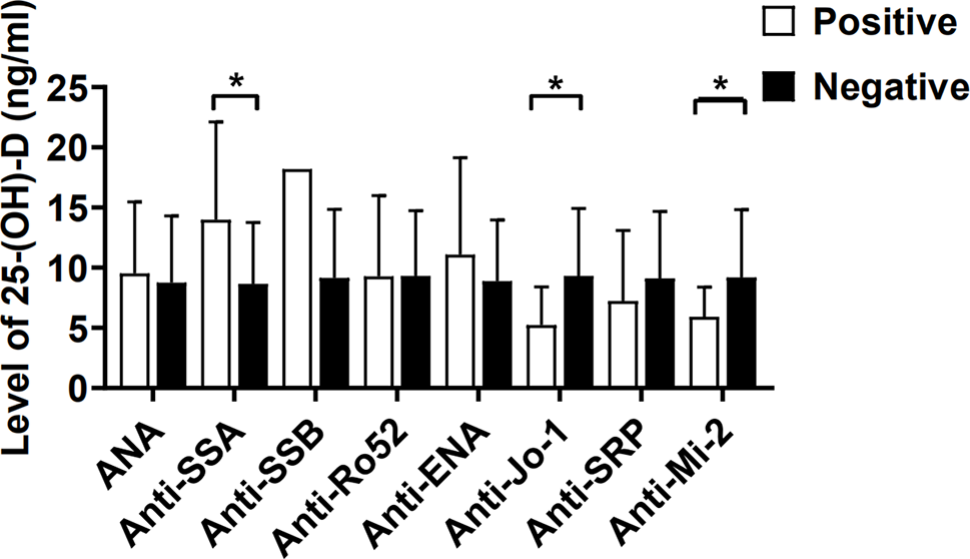
The serum levels of 25-(OH)-D (ng/ml) in IIM patients were compared between autoantibody positive and negative. ANA, Antinuclear antibody; SSA, Sjögren’s syndrome antigen A; SSB, Sjögren’s syndrome antigen B; Anti-Jo-1, anti-histidyl-tRNA synthetase; SRP, signal recognition particle. *p < 0.05.

The serum levels of 25-(OH)-D for IIM patients in the presence of anti-Jo-1 (5.24 ± 3.17 ng/ml) or anti-Mi-2 (5.93 ± 2.47 ng/ml) were significantly lower than those in the absence of anti-Jo-1 (9.32 ± 5.60 ng/ml; p = 0.037) or anti-Mi-2 patients (9.79 ± 5.68 ng/ml; p = 0.045) (**Figure 3**). Inversely, we also found that IIM patients with anti-SSA antibody positivity had higher levels of 25-(OH)-D than those in the negative patients (13.98 ± 8.12 vs 8.65 ± 5.12 ng/ml; p = 0.021; **Figure 3**).

**Figure 4:**
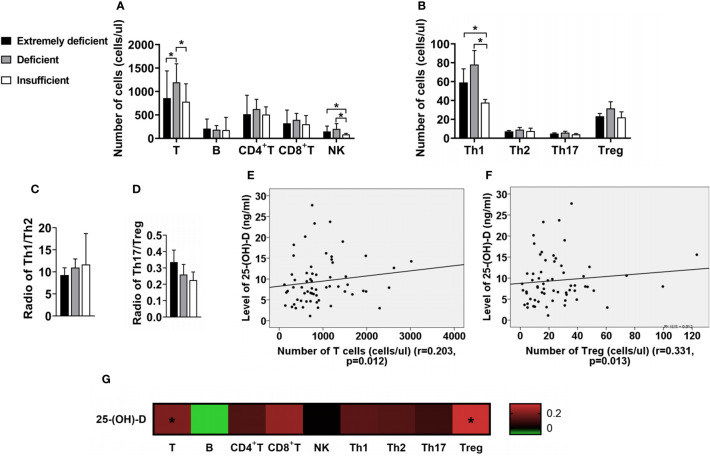
The changes of lymphocyte subsets in IIM patients with different vitamin D levels. **(A)** Numbers of T, B, CD4+T, CD8+T and NK cells. **(B)** Numbers of Th1, Th2, Th17 and Treg cells. **(C, D)** Radio of Th1/Th2 and Th17/Treg. **(E, F)** The serum 25-(OH)-D levels were positively associated with total T and Treg cells. **(G)** The associations of the levels of 25-(OH)-D with lymphocyte subsets in IIM patients. *p < 0.05.

The absolute number of total T, NK and Th1 cells of IIM with deficient vitamin D was significantly higher than those in IIM with insufficient vitamin D (**Figures 4A, B**). The ratios of Th1/Th2 or Th17/Treg had no significant difference (**Figures 4C, D**). However, the serum 25-(OH)-D levels were positively associated with total T (r = 0.203, p = 0.012; **Figure 4E**) and Treg cells (r = 0.331, p = 0.013; **Figure 4F**). No significant association was found between 25-(OH)-D and B, CD4+T, CD8+T, NK, Th1, Th2, Th17 cells or Th17/Treg (all p>0.05; **Figure 4G**). In addition, we found that IIM patients, who had lower levels of vitamin D, more often had heliotrope, gastrointestinal and liver involvement (**Table 3**). Interesting, we found that the level of Vitamin D was positively correlated with muscle strength and functional. The IIM patients has severer muscle weakness with lower vitamin D level (data not shown).

**Table 3:** Clinical features of IIM patients with extremely deficient, deficient and insufficient serum levels of 25-(OH)-D.

	Extremely deficient (N=42) %(n/N)	Deficient (N=17) %(n/N)	Insufficient (N=4) %(n/N)	*p*
Heliotrope	52% (22/42)	18% (3/17)	50% (2/4)	**0.049**
Gottron’s papules	38% (16/42)	24% (4/17)	25% (1/4)	0.525
Mechanic’s hands	14% (6/42)	6% (1/17)	0% (0/4)	0.497
Infections	60% (25/42)	71% (12/17)	50% (2/4)	0.642
Lung involvement	36% (15/42)	24% (4/17)	50% (2/4)	0.511
Cardiac involvement	31% (13/42)	47% (8/17)	50% (2/4)	0.430
Gastrointestinal involvement	21% (9/42)	0% (0/17)	0% (0/4)	**0.045**
Liver involvement	29% (12/42)	65% (11/17)	25% (1/4)	**0.030**
Kidney involvement	10% (4/42)	6% (1/17)	0% (0/4)	0.745

Results are given as percentage (count/total). Bold values mean statistical significance.

## Discussion

In this study, our data showed that newly diagnosed patients with idiopathic inflammatory myopathy (IIM) had significantly lower levels of serum 25-(OH)-D collected during the same month of the year compared with age- and sex-matched healthy controls, which was same with the results of Payam Azali et al. (6). Furthermore, there was no significant difference in the serum vitamin D levels between male and female in the IIM patients. There were 22 (40%) healthy volunteers with insufficient vitamin D levels, which is suggested that vitamin D deficiency was also prevalent in healthy adult. It has been reported that vitamin D deficiency related with several autoimmune diseases, including rheumatoid arthritis (RA) (3), multiple sclerosis (MS) (4), and systemic lupus erythematosus (SLE) (5). Now, our results suggested that low levels of vitamin D might be a risk factor for IIM.

We also found that the serum levels of 25-(OH)-D had no association with age in IIM patients, but they were positively associated with disease duration. Interestingly, Sahebari (10) had found similar results in RA, that 25-(OH)-D serum values in early RA were lower than those in the established RA. Sahebari thought that the higher serum levels of 25-(OH)-D in patients with established RA than in those with early RA could possibly be explained by the administration of a calcium vitamin D supplement together with other treatment. In our study, IIM patients were all untreated, so our explanation for the low levels of vitamin D in the patients with shorter disease duration is that the patients might be disabled and difficult in being outdoors because of musculoskeletal symptoms (9).

In IIM, serum inflammatory biomarkers such as ESR and CRP can be increased during the active phase of the disease (11). It has been found that patients with deficient in vitamin D had higher ESR and CRP values in RA (12) and IBD (13), but there was no similar report in IIM. In this study, our data showed that the CRP in IIM with deficient vitamin D was significantly higher than those in IIM with insufficient vitamin D. Another important feature of IIM patients was elevated serum muscle enzymes, which were the hallmark of muscle involvement. CK is released in the serum in case of muscle damage and is the most sensitive muscle enzyme in the acute phase of the disease (11). In DM or PM patients, CK concentrations are usually very high, often exceeding 10 times the upper limit of normal and sometimes as high as 50 times. CK concentrations in IMNM patients tended to be higher than those in DM and PM, while in IBM patients, these were generally lower, usually no more than 12 times the upper limit of normal values (14). In this study, in order to explore whether there is a correlation between vitamin D and muscle enzyme levels such as CK, we compared muscle enzyme levels in different vitamin D levels and analyzed the association between vitamin D and muscle enzyme levels. The data showed that as the degree of vitamin D deficiency increased, the levels of serum muscle enzymes elevated. In addition, a significant negative association between levels of 25-(OH)-D and ALT or AST was observed. Maybe vitamin D affects the severity of disease in IIM patients. These findings of our study are in agreement with those of Azimeh et al. (15). Furthermore, Hamidreza (16) found that high dose vitamin D supplementation was associated with a reduction in serum ALT, AST and LDH in people who had abnormal enzyme levels. Maybe this is the reason why IIM patients with lower levels of vitamin D were more often had liver involvement (**Table 3**).

There are two groups of autoantibodies in patients with IIM, myositis specific autoantibodies (MSAs) and myositis associated autoantibodies (MAAs) (17). MSAs are usually or exclusively found in DM/PM, while MAAs are usually found in mixed connective-tissue diseases (CTDs) with myositis. Our data showed that vitamin D deficiency was associated with anti-Jo-1 and anti-Mi-2 antibody in IIM (**Figure 3**). In IIM, the correlation between antibody titer and disease activity, pointed toward a pathogenic effect of the MSAs (17). Vitamin D may affect PM/DM by regulating the expression of MSAs, such as anti-Jo-1 antibody and anti-Mi-2 antibody.

The role of Treg cells in immune regulation has been widely confirmed. Many studies had found that functional deficiency of Treg cells existed in all subtypes of IIM (18–20), including the decrease of absolution numbers and the levels of related cytokines, which were considered as an important factor in the pathogenesis. Allenbach (21) found that in the mouse model of Experimental Autoimmune Myositis (EAM), depletion of Treg cells aggravated the disease, while injection of polyclonal Treg cells reduced both the incidence and the severity of myositis, further confirming the role of Treg cells in the pathogenesis of IIM. The reason for decrease of Treg cells in IIM patients is unknown. Carren et al. (22) found that 1,25(OH)_2_D_3_ can induce the generation of CD4^+^CD25^+^Foxp3^+^Treg cells, and down-regulate the generation of IL-17, IL-23 and other cytokines. In contrast, the number of Treg cells decreased when 1,25(OH)_2_D_3_ was deficient (23), suggesting that 1,25(OH)_2_D_3_ may play a significant role in the generation of Treg cells. In this study, although there was no significant difference in the absolute count of Treg cells in IIM patients with different vitamin D levels, the serum 25-(OH)-D levels were positively associated with the levels of Treg cells, which also proved this conclusion. We thought that decreased serum vitamin D levels in IIM patients led to functional deficit of Treg cells, finally causing the progression of disease. The possible mechanism considered that activating the vitamin D pathway could be a mechanism to decrease STAT1 and 3 activation and inflammatory cytokine output (24), and then affect the functional of Treg cells (25).

Our study also has some defects. First of all, the morbidity rate of IIM is low. To make a clear judgement, we need to set strict inclusion criteria; we studied the newly diagnosed and untreated IIM patients. So, the included cases were fewer. Secondly, patients were selected from the Shanxi Medical University Second Affiliated Hospital. At the same time, we used the classification criteria of Bohan and Peter, which was widely used by most clinical doctors in China, though it was somewhat outdated. Thus, these findings may not represent people in other areas. Then, since intake of vitamin D was characterized by various factors, such as different latitude area, frequency of outdoor activity, sun radiation, and other seasons, We did not rigorously balance factors in selecting patients for the study. So, We need a longer follow-up to determine whether vitamin D or analogues supplementation have therapeutic effects that improved the clinical symptoms and immune function of IIM patients.

In conclusion, vitamin D deficiency is common in IIM patients, which is significantly correlated with muscle enzyme, presence of anti-Jo-1 and anti-Mi-2 antibody, and the levels of total T cell and Treg in IIM. The patients with vitamin D deficiency were more likely to have heliotrope, gastrointestinal and liver involvement. It is suggested that vitamin D may play an important role in the immunological pathogenesis of IIM. Although our study exists some limits, it provides guidance for future studies if vitamin D could be a potential target in the prevention and treatment of IIM.

## Data Availability Statement

The original contributions presented in the study are included in the article/supplementary material. Further inquiries can be directed to the corresponding author.

## Ethics Statement

This study was approved by the Ethics Committee of the Second Hospital of Shanxi Medical University and all patients have signed informed consent.

## Author Contributions

ZY performed the data analyses and wrote the manuscript. HC, YL, TD and CY participated in the collection of samples and clinical data. CG participated in the study design and revising of the manuscript. HW provided intellectual input and supervision throughout the study and made a substantial contribution to manuscript drafting. All authors contributed to the article and approved the submitted version.

## Conflict of Interest

The authors declare that the research was conducted in the absence of any commercial or financial relationships that could be construed as a potential conflict of interest.
